# Advances and Perspectives for Polyploidy Breeding in Orchids

**DOI:** 10.3390/plants11111421

**Published:** 2022-05-27

**Authors:** Pablo Bolaños-Villegas, Fure-Chyi Chen

**Affiliations:** 1 Fabio Baudrit Agricultural Research Station, University of Costa Rica, La Garita District, Alajuela 20101, Costa Rica; 2 Lankester Botanical Garden, University of Costa Rica, Dulce Nombre District, Cartago 30109, Costa Rica; 3 Faculty of Food and Agricultural Sciences, Rodrigo Facio Campus, School of Agronomy, University of Costa Rica, Montes de Oca County, San Jose 11503, Costa Rica; 4 General Research Service Center, National Pingtung University of Science and Technology, #1 Shuefu Road, Neipu township, Pingtung 91201, Taiwan; fchen@mail.npust.edu.tw

**Keywords:** orchid breeding, polyploidy, meiosis, fertility, flower size

## Abstract

The orchid market is a dynamic horticultural business in which novelty and beauty command high prices. The two main interests are the development of flowers, from the miniature to the large and showy, and their fragrance. Overall organ size might be modified by doubling the chromosome number, which can be accomplished by careful study of meiotic chromosome disjunction in hybrids or species. Meiosis is the process in which diploid (2n) pollen mother cells recombine their DNA sequences and then undergo two rounds of division to give rise to four haploid (n) cells. Thus, by interfering in chromosome segregation, one can induce the development of diploid recombinant cells, called unreduced gametes. These unreduced gametes may be used for breeding polyploid progenies with enhanced fertility and large flower size. This review provides an overview of developments in orchid polyploidy breeding placed in the large context of meiotic chromosome segregation in the model plants *Arabidopsis thaliana* and *Brassica napus* to facilitate molecular translational research and horticultural innovation.

## 1. Introduction

**Figure 1:**
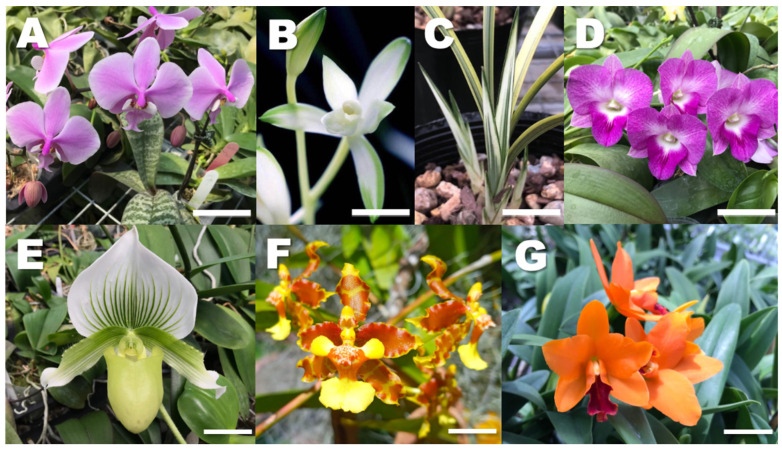
Representative contemporary orchid hybrids and species. *Phalaenopsis schilleriana* (**A**), a popular diploid species (2n = 38) used as parent in tetraploid hybrids, *Cymbidium ensifolium* ‘Variegata’, flowers (**B**), and variegated leaves (**C**), a prized diploid species (2n = 40) revered in traditional Chinese art, *Dendrobium phalaenopsis* (**D**), a diploid species (2n = 38) who may have crossed via unreduced gametes to *D. discolor* and thus produced the natural triploid hybrid *D. superbens* (3n = 54), *Paphiopedilum* Maudiae (**E**), a natural interspecific hybrid between *P. callosum* and *P. lawrenceanum* believed to be a tetraploid with a variable chromosome number (4n = 33–35), *Oncidium stenotis* (**G**), a species (probably diploid, 2n = 42) with large flowers and long stems, and with great horticultural potential *Rhyncattleanthe* Young-Min Gold ‘Golden Diamond’ (**G**), an intergeneric hybrid between *Rhyncholaelia* Schltr., *Cattleya* Lindl., and *Guarianthe* Dressler, and probably a diploid (2n = 40). Approximate scale bar: 2.5 cm (**A**,**C**–**E**,**G**), 1 cm (**B**,**F**). Photos taken by F. C. Chen (**A**,**D**,**E**,**G**) in Pingtung County, by Amigo Hsieh (**B**,**C**), in Tainan County, and by P. Bolaños-Villegas (**F**) at the Lankester Garden in Cartago, all in 2022.

Orchids, including *Phalaenopsis* (2n = 2x = 38) [1] (Figure 1A), also called the moth orchid, are among the most popular ornamental potted plants currently traded around the world [2]. Other famous genera traded are *Cymbidium*, the Chinese ‘*lan*’ orchid (2n = 2x = 40) [3] (Figure 1B and C); *Dendrobium* (2n = 2x = 38) [4] (Figure 1D); *Paphiopedilum* (2n = 2x = 26), the famous lady slippers [5] (Figure 1E); *Oncidium* (section *Oncidium*, 2n = 2x = 42), the dancing-lady orchids [6] (Figure 1F); and *Cattleya* (2n = 2x = 40) [7,8] (Figure 1G), among many others.

Rational breeding of commercial orchid cultivars may have been initiated in 1896 in Hawaii with the introduction of *Dendrobium superbum* from the Philippines [4], followed by a formal research program initiated at the University of Hawaii in 1950 that involved a cooperation with the University of Hiroshima in Japan [4,9]. This program focused on cytogenetics, cross-compatibility, and meiotic behavior, and quickly concluded that polyploidy is key to breeding superior hybrids in *Dendrobium* and *Vanda* [4,9] while also establishing that genome homology is crucial to breeding *Phalaenopsis* [10]. Since the 1980s, large-scale orchid production has shifted to Taiwan and has been accompanied by research into *in vitro* seed germination, micropropagation, and the study of meiosis and pollination to overcome hybridization barriers, especially in *Dendrobium* and *Phalaenopsis* [11]. This is a serious horticultural operation worth 200 million USD per year in Taiwan and about 85 million USD in Thailand [12].

To remain competitive, new orchid cultivars must be developed continuously to meet the market demands [6]. Usually, both wild species and commercial cultivars are chosen as parent plants in breeding programs that rely on interspecific hybridization and selection of progenies [2] because sexual reproduction may increase heterosis (see Table 1) and diversity of traits [3], including flower longevity [7]. Nonetheless, high infertility is often the result of breeding these advanced orchid hybrids [13]. It is believed that about 60% of pollination events fail to induce fruit development in most orchid genera [11]. The reported causes are many, varying from untimely tapetal degeneration in *Oncidesa* [6] to premature chromatid separation and formation of lagging chromosomes during metaphase I in *Aranda* [14]. Misplaced bivalents with no clear position along the spindle, also called pseudo-bivalents, have also been reported in *Vanda* semi-terete diploid hybrids [9]. Traditionally, fertility can be restored in these orchid hybrids by doubling the number of chromosomes with antimitotic agents such as colchicine and oryzalin during tissue culture [13]; the result is an allotetraploid [15] (see Table 1). Ideally, the process induces disomic pairing of homologous chromosomes during meiosis I and the creation of balanced gametes [13], but unfortunately, chromosomes may undergo structural rearrangements under the influence of colchicine, which may reduce fertility [16].

Polyploid orchids, including autopolyploids (which arise from a single species) (see Table 1) have desirable traits such as large flowers with great substance, round conformation and intense coloration, and thicker stems and leaves [13]. In *Cymbidium*, newly synthesized sexual allopolyploids (see Table 1) such as ‘Yutao’ show increased width and thickness of sepal, petal, and lips, and flowers are rounder and produce more fragrance; therefore, they have increased commercial value [3]. These polyploids are bred from naturally produced unreduced gametes (see Table 1), and these plants are considered better than those obtained from somatic polyploidization because of the resulting genetic diversity and heterosis [3]. As in the case of *Dendrobium* (see Figure 1A), the horticultural value of these unreduced gametes is enhanced by the following facts [4]: (1) pollination of a single orchid flower can potentially produce half a million to a million seeds, so a hybrid with 1% fertility can give rise to 5000 to 10,000 viable seeds, and fertility as low as 0.01% may still produce 50 to 100 viable seeds; (2) endosperm tissue is lacking in most seeds, so embryo–endosperm antagonism does not exist; and (3) orchid embryos are cultured in aseptic nutrient culture, which allows for better survival of hybrids as compared with the normal seeding of other plants.

Evolutionarily speaking, polyploid organisms are often more resilient to extreme environments and cataclysms because of their increased genetic variation and the buffering effect of their duplicated genes [21,26]. For instance, whole genome duplications (WGDs) (see Table 1) may have contributed to gene diversification and fine-tuning of orchid ovule initiation and closure of the stigmatic cavity, as in the case of the *DROOPING LEAF/CRABS CLAW* (*DL/CRC*)-like genes in *Phalaenopsis equestris* and *Dendrobium catenatum* [27]. Additionally, in the genome of *P. equestris*, a large WGD event was associated with the Cretaceous-Paleogene extinction about 66 million years ago. This WGD is believed to have been followed by intense radiation that enabled the *Orchidaceae* to become the second largest angiosperm plant family with its remarkable diversity in flower morphology [28].

In nature, orchid autotetraploids (see Table 1) of *Gymnadenia conopsea* exhibit high pollination and fruiting success [29], possibly because of changes in flower scent, changes in pollinaria morphology, larger flowers, and low inbreeding depression [29]. Thus, a higher reproductive fitness of tetraploids than diploids may enable them to become more common [29]. In fact, genome duplication events are assumed to be beneficial for orchid breeders in search of new morpho-types and improvements in size, substance, and form [30].

This review gives an overview of meiotic mechanisms and breeding techniques that allow for polyploidization of plant gametes to highlight opportunities for molecular breeding in orchids for the accelerated creation of a new generation of elite hybrids.

## 2. Polyploidy Breeding in Orchids

Allopolyploids are usually created by hybridizing related species (allo = different); thus, the resulting individual may have divergent genomes combined within its own chromosome complement [31]. Merging genomes from different species provides genome variation and novel opportunities to diversify, with the added advantage that gene redundancy may mask recessive deleterious alleles by dominant ones [31]. Additionally, the expression of genes required for chromatid cohesion and meiosis may be enhanced, as observed in the *Arabidopsis suecica* allopolyploid [22]. The relative abundance of meiotic multivalents (more than three chromosome pairs) (see Table 1) has been used as a cytological factor to distinguish auto- and allopolyploids. For instance, the prevalence of multivalent pairing at metaphase I may point to homology between chromosome sets and thus autopolyploidy [32]. However, in contrast, a high formation of bivalents (see Table 1) at diakinesis may result from pairing between non-homologous (homoeologous) (see Table 1) parental chromosome sets, which may indicate allopolyploidy [32], although this behavior is not absolute [32]. In the actual case of orchid breeding, the development of cultivars with multiple spikes involves crossing species such as *Phalaenopsis micholitzii* (with multiple short spikes) and *Phalaenopsis tetraspis* (long spikes) [33]. The resulting hybrid, *P.* Tzu-Chiang Tetralitz, develops up to five spikes [33]. In many cases, tissue culture (see Table 1) induces spontaneous polyploidization of hybrids [33], but selection is laborious, and crosses must be redone often to retain stability of traits in the progenies. Early generations of synthetic allopolyploids show quick and broad reorganization of the merged genomes, including chromosome rearrangements and changes in chromosome number as well as epigenetic modifications, such as transposon activation, chromatin modifications, and altered methylation patterning [32]. Indeed, chromosome rearrangements are often observed in meiocytes of presumptive orchid allopolyploids [34], along with micronuclei (see Table 1) in tetrads. These micronuclei are common in human cancer cells and arise from hypomethylation in peri-centromeric DNA, dysfunctional kinetochore assembly, poor organization of the spindle, or uncoordinated expression of anaphase checkpoint genes [35].

## 3. Chromosomal Pathways for Meiotic Polyploidization

Although several reproductive mechanisms may create polyploid plants, most plants are formed by the random production of diploid (2n) gametes [36]. However, despite the huge biological and agricultural significance of forming diploid gametes, the molecular mechanisms that lead to the formation are not well understood [36]. Cytologically, 2n gametes possess the somatic chromosome number because of meiotic defects, which leads to a *mitosis-like*/*non-reduced* division with dyads (2n) (see Table 1) formed alongside triads (3n) and normal haploid tetrads (n) [37] (see Table 1), a phenomenon called meiotic nuclear restitution [37]. Most of these 2n gametes result from a few basic processes of nuclear restitution: (1) omission of meiosis I (also called first division restitution [FDR]) (see Table 1), (2) omission of meiosis II (also called second division restitution [SDR]) (see Table 1), (3) defects in spindle organization and (4) incomplete cytokinesis [37,38] (see Table 1).

In eukaryotes, meiotic cell division halves the chromosome number in gametes via a single DNA replication followed by two events of chromosome segregation [39]. During meiosis I, homologous chromosomes should pair and synapse and exchange genetic information via recombination. Then the crossover sites, called chiasmata (see Table 1), form physical links between the two homologs and ensure (1) proper placement of the bivalent in the spindle and (2) proper segregation of homologs during anaphase I [40]. To achieve this, sister kinetochores from each homolog attach to microtubules extruding from the same spindle pole (e.g., monopolar kinetochore attachment), and cohesion is removed at the chromosome arms but not centromeres. Then during meiosis II, chromosome segregation in the two new haploid nuclei proceeds in an equational fashion. Chromatid centromeres are attached bipolarly to the microtubules up to anaphase II, when cohesion is finally removed to ensure that chromatids segregate into four haploid daughter cells [40].

## 4. Synapsis, Chromosome Segregation and Meiotic Non-Reduction

Meiotic division and gametophytic ploidy (see Table 1) are tightly regulated processes at the molecular level, and many of these regulators have been successfully characterized [38]. For instance, mutations in the *Arabidopsis thaliana* gene *DYAD/SWITCH1* (*SWI1*; *At5g51330*) (see Table 2) functioning in cohesion regulation [41] and its maize and rice homologs, both named *AMEIOTIC1* (*GRMZM5G883855* and *Os03g44760*) [42], lead to the abrogation of synapsis during meiosis I and rather turn it into a mitotic-like cycle [43]. However, in the *Arabidopsis* mutants *parallel-spindle 1* or *Jason*, disturbed orientation of the spindle leads to a similar effect [43]. These features are often referred to as examples of FDR [43] and are somewhat common in amphihaploid- (see Table 1) and polyhaploid-wide (see Table 1) F_1_ hybrids in which homology is very low and meiotic pairing does not occur [39]. Thus, some researchers consider that this type of meiotic non-reduction is better described as asynaptic- or univalent-dependent [39].

And what is synapsis? At the early stages of meiosis, homologous chromosomes find each other within the tight confines of the nucleus and then become fully aligned in a process called homologous chromosome pairing [50]. Once chromosome stretches are paired, those same regions will be held together by a scaffold of proteins called the synaptonemal complex (SC). This tight alignment is called synapsis and allows for recombination, in which information is exchanged between the parental homologs and generates crossovers that promote faithful chromosome segregation during meiosis I [50]. *Arabidopsis* genes that regulate establishment of the cohesion (e.g., the entrapment of DNA) between homologous chromosomes can affect the assembly of synaptonemal complex. Such genes include *STRUCTURAL MAINTENANCE OF CHROMOSOMES 5* (*SMC5*; *At5g15920*), *STRUCTURAL MAINTENANCE OF CHROMOSOMES 6 A/B* (*SMC6A/B*; *At5g07660*, *At5g61460*) and *PRECOCIOUS DISSOCIATION OF SISTERS 5* (*PDS5A*/*E*; *At5g47690*, *At1g77600*, *At4g31880*, *At1g80810*, and *At1g15940*). However, whether these mutants show signs of FDR during meiosis is unclear. Another gene that regulates synapsis and cohesion is *SYNAPTIC1*, also known as *RECOMBINATION 8/SYNAPTIC 1* (*REC8*/*SYN1*; *At5g05490*) [49] (see Table 1). This gene has a severe impact on meiotic synapsis by affecting the correct polymerization of the SC [43], and its absence during meiosis I leads to illegitimate interhomolog recombination and catastrophic chromosome fragmentation [43]. Univalents are also produced owing to failure to synapse, but their chromosomes are extremely tangled, and distinguishing between them is difficult [51]. Thus, because the phenotype is so extreme, it might be necessary to develop weak alleles to induce an FDR-like phenotype that might be useful for orchid breeding.

For the formation of 2n gametes through SDR, several *Arabidopsis* genes have been linked to the omission of meiosis II, including *OMISSION OF SECOND DIVISION 1* (*OSD1; At3g57860,* also known as *GIGAS* and *UVI4-Like*) (see Table 2), a key negative regulator of the anaphase-promoting complex/cyclosome (APC/C) that may control the turnover of cyclins to elicit the exit from mitosis or meiosis [45,52]. Another gene is the plant A-type cyclin gene *CYCA1;2*/*TARDY ASYNCHRONOUS MEIOSIS* (*TAM; At1g77390*) (see Table 2), which is essential for the transition between the first and second meiotic division and whose mutations cause exit from meiosis after prophase [45]. In Arabidopsis, mutants for *TAM* develop diploid gametes [45], whereas mutants for *OSD1* develop triploid or tetraploid gametes [45]. Combining both mutations led to the production of tetraploid spores, and by adding mutant alleles for meiotic recombination (*spo11-1*) and for segregation (*rec8*/*syn1*), meiosis is completely abrogated in the *tam* or *osd1* backgrounds. This results in the now legendary *Mitosis into Meiosis* (*MiMe*) phenotype [45] that was introduced in rice to produce apomictic progenies identical to the mother plant [53]. This might be an interesting approach to mass-reproduce valuable orchid hybrids, perhaps by CRISPR-Cas–mediated transformation, as was shown recently in Taiwan with the orchid model species *P. equestris* [54]. Moreover, the *Orchidstra 2.0* transcriptomic database developed by the Academia Sinica (Taipei) allows for identifying orthologues for up to 17 orchid species, including *P. equestris* [55]. Hence, it might be technically feasible to perform genome editing and test induction of unreduced gamete formation or *MiMe*-like phenotypes.

A third gene that is epistatic to both *OSD1* and *TAM* is *SUPPRESSOR WITH MORPHOGENETIC EFFECTS ON GENITALIA7* (*SMG7*; *At5g19400*) [56]. This gene may operate as a regulator of the first to second meiotic division transition, probably by down-regulating or inducing the degradation of *CDKA;1* [56]. Pollen mother cells in the *smg7* mutants that arrest during anaphase II do not seem to form any pollen, as seen by Alexander Red staining [56]. Perhaps yet-identified alleles of interest may be present in orchids.

## 5. Cytokinesis, Heat Shock and Polyploidy

Cytokinesis is another key cellular process that may lead to the production of polyploid gametes [38]. In plants, meiotic cytokinesis is timed to occur after chromosome segregation, but the process varies somewhat between dicots and monocots [46]. In dicots such as *Arabidopsis*, meiotic cell walls are synthesized after the separation of the sister chromatids, which occurs at the final stages of meiosis II, called simultaneous cytokinesis [46]. Nevertheless, in monocots such as rice and maize, meiotic cytokinesis involves the synthesis of a cell wall after each round of chromosome segregation. Hence, a dyad is formed at the end of meiosis I, whereas tetrads are formed after meiosis II [46]. This type is called successive cytokinesis. Mitogen-activated protein kinases (MAPKs) are common signal transduction factors that control meiotic cytokinesis [38], and they operate in a relay fashion, a signaling cascade that may involve MAPK kinase kinases, MAPK kinases (MKKs), and MAPKs, which are activated sequentially by phosphorylation at conserved activation sites [57]. Of these, the interaction between *Arabidopsis NPK1-ACTIVATING KINESIN 2*/*TETRASPORE* (*NACK2*/*TES*, *At3g43210*), *NPK1-RELATED PROTEIN KINASE 3* (*ANP3*, *At3g06030*), *MKK6* (*At5g56580*), and *MAPK4* (*At4g01370*) (see Table 2) have been shown to mediate male meiotic cytokinesis [46,57]. In the case of the *tes* mutants, all tetrads share the same cytoplasm and initiate male gametogenesis together, thus leading to the formation polyploid sperm, as seen by DAPI staining [48]. This situation is presumably due to defects in the assembly of the radial microtubule system (also called the phragmoplast), which leads to severe microtubule accumulation in nuclear surfaces and total failure to establish cytoplasmic domains [48]. Of note, mutants for suppressors of gibberellin signaling, specifically *rga-24* and *gait6*, show similar alterations at telophase II, thus leading to the formation of diploid gametes in *Arabidopsis* (3.3%). Spraying with 100 µM GA_3_ caused the same phenotype (3%) in the *Ler* phenotype [38].

Work in *A. thaliana* and *Brassica napus* has shown that heat, cold, and drought stress may affect the expression or activity of MAPKs [57]. Remarkably, in *Arabidopsis,* heat stress (36–38 °C for 24 h) caused defects in cytokinesis at metaphase I, namely reduced abundance of microtubule fibers, failure to form a bipolar spindle, formation of multiple mini-phragmoplasts at anaphase I, and total failure to form a radial microtubule system at the tetrad stage [46]. Moreover, fluorescent *in situ* hybridization with a centromere probe suggested that during meiosis I, homologous recombination or crossover formation is impaired, because only univalents are observed [46] (see Table 1).

Cold stress is effective in inducing the formation of 2n gametes in *Arabidopsis* [36]. With cold shock treatment of 4 to 5 °C for up to 40 min followed by sampling seven days later, 6% to 38% of flowers showed enlarged pollen grains. The sperm nuclei in these pollen grains consistently showed extra centromere foci, as seen by expression of the *pWOX2:CENH3:GFP* centromeric reporter construct, thus suggesting the formation of diploid, triploid, and tetraploid male spores [36]. Results indicate defects in the formation of cell plates between tetrads at telophase II, which suggests that these are recombinant, SDR-type unreduced gametes [36]. Perhaps this approach could be used in orchid breeding programs as well.

## 6. Recombination, Heat Shock and Polyploidy

The relation between heat stress (36–38 °C for 24 h) and reduced meiotic recombination has been explored in *A. thaliana* by immunostaining and transcriptional analyses [44]. Apparently, assembly of the synaptonemal complex is disturbed, as seen by changes in the distribution and abundance of the chromosome axis protein ASYNAPTIC1 (ASY1, At1g67370) (see Table 2) and lateral element/transverse filament protein ZYP1A (At1g22260) (see Table 2) and by the absence of bivalents at metaphase I. The expression of recombinase *RAD51* (*At5g20850*) is also reduced, which suggests impaired processing of double strand breaks usually implemented by the conserved type-II topoisomerase SPO11 (At3g13170) [44]. Abnormal tetrads were observed, including what appeared to be dyads [44]. Thus, taken together, heat stress appears to be valuable for the formation of unreduced-like gametes, probably nearly FDR-type ones. A potential caveat is that ZYP1A is also involved in the formation of class I interfering crossovers [58], and in its absence, crossover formation is not prevented but rather promoted, as seen by an increase of up to 50% in levels of the crossover makers HEI10 and MLH1 [59].

Heat stress (34 °C vs. 21 °C, for up to one week) has been shown to shorten meiosis in *Arabidopsis* from 21.1 h at 21 °C to 18.1 h at 34 °C [60]. Ingeniously combining time-lapse analyses of microtubule organization (*TagRFP-TUA5*), breakage of the nuclear envelope (NEB), localization of CDKA;1-stress granules (*CDKA;1-mVenus*) and assembly of the SC in chromosomes (*ASY1-RFP* and *ZYP1b-GFP*) concluded that during zygotene, ASY1 is unexpectedly depleted and the loading of ZYP1 is aborted abruptly. The abortion is possibly due to activation during pachytene of a newly discovered plant cell cycle checkpoint controlled by the kinase ATAXIA TELANGIECTASIA MUTATED (ATM; At3g48190) [60]. In these heat-stressed meiocytes, homologs fail to pair properly, and chromosome bridges and fragments are observed, possibly caused by non-homologous recombination [60]. The value of this type of work in the context of orchid breeding is that the expression of these constructs could be attempted by *Agrobacterium*-mediated transformation in *Phalaenopsis,* as is done in Taiwan [54] for the analysis of meiosis in response to all types of experimental conditions.

## 7. Polyploidization and Recombination

**Figure 2:**
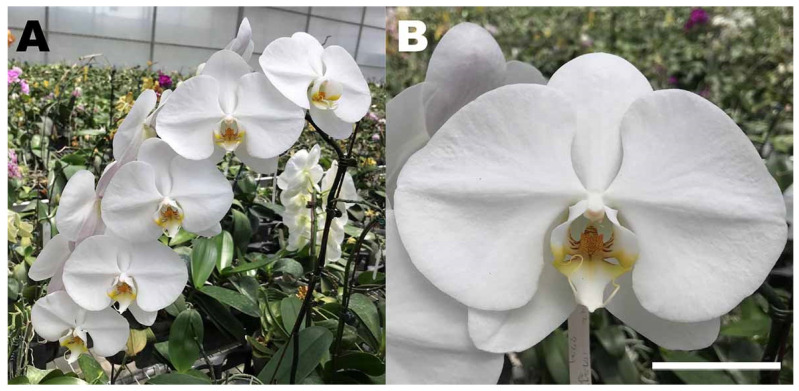
Flower spike (**A**) and individual flower (**B**) in elite tetraploid hybrid *Phalaenopsis* Sogo Yukidian ‘V3’ (2n = 4x = 76), the standard white hybrid most sold in Taiwan. This extremely stable meiotic hybrid was derived from *Phalaenopsis* Doris, an old interspecific hybrid synthesized from crossing *Phalaenopsis amabilis*, *Phalaenopsis rimestadiana* and *Phalaenopsis aphrodite*. The spikes carry multiple flowers all evenly spaced, and the flowers themselves are symmetrical, large, flat, and long lived. Scale bar: 2.5 cm. Photos taken by F.C. Chen in Pingtung County, 2022.

In *Phalaenopsis* orchids, species and primary F_1_ hybrids are usually diploid, whereas elite varieties are tetraploid [61]; examples are *Phalaenopsis* Sogo Yukidian ‘V3’ (see Figure 2A,B), *Phalaenopsis* Tai Lin Red Angel ‘V31’ and *Phalaenopsis* Brother Irene ‘Feng Fong’. All have 2n = 4x = 76 chromosomes [62]. Such parental varieties are sexually crossed with other varieties and species to introgress traits such as quick growth, ease of clonal reproduction, fragrance, and resistance to disease [62]. Thus, enhanced sexual recombination might constitute a valuable horticultural asset.

Work in *Brassica* allotriploids (AAC genome, 2n = 3x = 29) derived from the species *Brassica rapa* (AA genome, 2n = 2x = 20) and *Brassica napus* (AACC genome, 2n = 4x = 38) has shown an increase in crossover formation (1.7 to 3.4 times), possibly corresponding to type I interfering crossovers [19]. This work involved the use of 199 single nucleotide polymorphisms (SNPs) evenly distributed across chromosomes and revealed that the increase in recombination is the highest in pollen-receiving plants, at long chromosomes and at pericentromeric regions [19].

Additionally, the combination of cytogenetics and SNP markers can assist in the identification of regulators for aberrant homoeologous recombination in genotypes that are suspected to be genetically unstable, as in the case of the *B. napus* double haploid population SGDH [63]. Partial homoeology has been suggested to cause meiotic irregularities and defective tetrad formation in orchids such as *Aranda* ‘Christine’ C80 [14], an old interspecific hybrid known for being genetically unstable [14]. Illegitimate recombination between homoeologues may cause aneuploidy (see Table 1) because homoeologous bivalents, multivalents and univalents may not segregate correctly at meiosis I [64]. Additionally, the uneven exchange can lead to gain or loss of homoeologous segments when chromatids segregate [63]. In *Brassica napus* SGDH plants, the formation of multivalents seems common, as observed by fluorescent *in situ* hybridization, and duplications and deletions are widespread, as confirmed using SNP markers. Most of this variation seems linked to the presence of a region of 10.3 to 23.9 Mbp nested within chromosome 9, named *BnaA9*, which shows a strong change in the expression of orthologues for *REPLICATION PROTEIN A 1C* (*RPA1C*; *At5g45400*) and *MMS and UV Sensitive 81* (*MUS81; At4g30870*) [63] (see Table 2). The endonuclease MUS81 is an important mediator in the resolution of recombination intermediates such as double Holliday junctions [64], whereas RPA1C is part of the heterotrimeric RPA complex that mediates activation of DNA damage checkpoints [65].

Thus, although in polyploid hybrids, it might be possible to fingerprint for alleles that might cause genetic instability, breeders might want to look for a long-term solution. Cytologically, in allo-haploid, tissue-cultured *Brassica napus* cv. *Tanto*, the reduction in copy number of *ARABIDOPSIS MUTS HOMOLOG 4* (*MSH4*; *At4g17380*) (see Table 2), by just one copy, effectively reduced the frequency of events of homoeologous recombination, possibly constituting an evolutionary mechanism for fine-tuning meiosis [47]. This gene is part of the main crossover pathway, called the ZMM pathway because it involves genes *ZIP1*, *MER3*, *MSH4*, *MSH5*, *SHOC1*, *HEI10*, and *PTD*. Remarkably, many of them (e.g., *MER3*, *MSH4* and *MSH5*) show rapid loss of duplicates following evolutionary events of WGD in angiosperms [47]. One might envision the use of CRISPR-Cas9 editing to trim the number of *MSH4* duplicates in valuable orchid polyploids to stabilize meiosis [16]. In fact, in allopolyploids, the ZMM pathway could be targeted to reduce the number of crossovers to the bare minimum, one per chromosome pair to prevent inter-homoeologue crossover formation [47].

## 8. Post-Polyploid Diploidization in Orchids

In the 1960s in Hawaii, allopolyploid *Vanda* hybrids showed preferential pairing of chromosomes during meiosis [9]. Then in the 1980s in Malaysia, the diploid species *Calanthe veratrifolia* showed evidence of asynchronous segregation of subgroups of chromosomes during meiosis, involving multiple spindles, perhaps suggesting a polyploid ancestry or concealed hybridity [66]. In those days, this behavior was called complement fractionation [66] (see Table 1). Currently this phenomenon is better known as post-polyploid diploidization (PPD) [21] (see Table 1), and it may have driven the evolution of paleo- and meso-polyploid lineages into diploid genomes following a WGD event. The process is thought to involve genome downsizing, subgenome-specific fractionation, and modulation of gene expression [21]. A key feature of genome fractionation during PPD is that one of the parental subgenomes generally retains significantly more genes as compared with the other subgenome, especially in the case of dosage-sensitive genes [67] (see Table 1). Such genes are involved in macromolecular complexes, transcription regulation and responses to environmental stimuli [23]. The determination of genome dominance (see Table 1) is suspected to be linked to differences in the density of transposable elements, methylation, and siRNA expression [23]. For instance, the subgenome “A” of *A. suecica* shows decreased levels of CG methylation and transcriptional up-regulation at loci required for proper chromatid alignment during meiosis, possibly as a mechanism to promote reproductive stability [22]. Example loci are homologs of *STRUCTURAL MAINTENANCE OF CHROMOSOMES 3/TITAN 7* (*SMC3/TTN7*; *At2g27170*), *SMC1/TTN8* (*At3g54670*), *SMC6B/MIM* (*At5g61460*) and *PDS5B* (*At1g77600*) (see Table 1).

At the cytological level, PPD may manifest in orchid hybrids as chromosomal rearrangements such as centric fissions, inversions and Robertsonian translocations between homologous and non-homologous chromosomes (see Table 1) that lead to speciation and diversification. Or if expressed alternatively, PPD may transform a polyploid genome into a quasi-diploid one with a lower base chromosome number (x) (e.g., descending disploidy) (see Table 1) that reverts the number of linkage groups to the same number as the diploid ancestors [21]. Inversion loops and translocation junctions have been reported at pachytene in *Doritaenopsis* Fuchsia Princess ‘KHM648’ (2x = 38) [1]. However, chromosomal bridges have been observed at anaphase I in chromosomes of *Dtps*. Sweet Strawberry ‘Wei’ (4x = 76) and *Dtps*. Ben Yu Star ‘Red Dragon’ (4x = 76) [34], both suggesting the existence of PPD in commercial hybrids of *Phalaenopsis*. Widespread variation in chromosome number in *Paphiopedilum* section *Barbata* is suspected to be caused by centric fissions (2n = 28, 30, 32, 33, 34, 35, 36, 37, 38, 40, 41, and 42) [20].

Although not entirely obvious, commercial hybrids may offer the opportunity to study orchid PPD in real time and in a controlled setting to facilitate understanding the molecular mechanisms that are believed to mediate PPD and genome fractionation, such as chromatin accessibility and histone modifications [67].

The experimental hurdle is that the *Orchidaceae* family (Figure 2) comprises more than 28,000 species and 736 genera and that the patterns of karyotype (see Table 1) evolution are not well understood [21]. What is clear is that the sequenced orchid genomes that are currently available (*Phalaenopsis equestris*, *Dendrobium catenatum*, *Dendrobium officinale* and *Apostaceae shenzhenica*) appear to have high chromosome numbers (2x = 38, 2x = 38, 2x = 38, and 2x = 68), which suggests paleo-polyploid origins [21,68]. Additionally, a huge disparity in orchid chromosome numbers, ranging from 12 in *Erycina pusilla* to 240 in *Epidendrum cinnabarinum*, indicates that many WGD events followed by diploidization may have taken place during evolution across different clades [21,69,70]. 

In contrast, by comparing the karyotypes of species in *Phalaenopsis* versus interspecific hybrids, one may deduce, in a short time, a few trends for PPD. For instance (1) during the selection of sexually fertile progenies in harlequin and novelty cultivars (*P*. Chian Xen Magpie, *P*. Chian Xen Piano ‘CX339’), there is a strong fractionation bias against large chromosomes from sections *Polychilos*, *Esmeraldae* and *Parishianae*; (2) in specific triploids such as *P*. Golden Sands ‘Canary’, *P*. Taipei Gold ‘STM’, *P*. Queen Beer ‘Mantefon’, *P*. Joy Spring Canary ‘Taipei’, *P*. Sogo Relax ‘Sogo F-987’ and *P*. Liu’s Berry ‘SW’, irregular pairing of chromosomes is common, presumably due to chromosomal rearrangements; and 3) the production of unreduced gametes has certainly led to the formation of valuable tetraploids such as *P*. Taipei Gold ‘Gold Star’ and *P.* Paifang’s Queen ‘Brother’ [71]. Finally, the implementation of genomic *in situ* hybridization may facilitate the identification of parental genomes for the introgression of key horticultural traits [71], and the identification of alleles for *OSD1* and *TAM* might accelerate the development of new elite polyploids [24].

## 9. Induction of Polyploidy on Meiocytes

A novel polyploid individual may form via different pathways and depending on the pathway and the level of heterozygosity, a newly formed polyploid will perform better, as shown by gains in growth, fertility, and horticultural quality [13,19]. This is important because horticulturally speaking, the current understanding of inter- and intrageneric polyploidization, and *in vitro* propagation in *Phalaenopsis*, *Epidendrum*, *Lycaste*, *Cymbidium,* and *Dendrobium* has been obtained from somatic chromosome doubling with colchicine in solid and liquid medium (0.01–0.005%) [25,61], oryzalin in liquid medium (57 µM) [13], and amiprophos-methyl (APM) in liquid medium (2.5 μM) [72] , followed by flow cytometry analysis and selection of progenies [72], which may show somaclonal variation [25]. However, this path does not reproduce what usually happens in nature because mitotic non-disjunction of sister chromatids in meristem tissues, zygotes or embryos is rare and unfortunately restricts the number of alleles fixed per locus in auto- and allotetraploids [19]. In contrast, meiotic chromosome doubling is known to result in larger genetic variability, fitness, and heterozygosity than somatic (mitotic) doubling [73].

In cultured *Cymbidium*, unreduced gametes form at a rate of 0.15% in the cultivar ‘Xiaoxiang’ to 4.03% in cultivar ‘47–17’, and the formation is believed to be the highest in interspecific hybrids [3]. However, in *Begonia*, the formation of 2n gametes can be boosted by treating pollen mother cells with trifluralin (10, 100 and 1000 µM in 5% DMSO for 24 h) and nitrous oxide (N_2_O) at 6 bar (600 kPa) for 48 h [73]. Additionally, in *Populus canescens*, the injection of colchicine (0.5% v/v) during pachytene leads to unreduced gamete production at an astonishing rate of 30%, with the germination rate not significantly affected (22% vs. 23% in natural unreduced gametes) [74].

Work in *Caenorhabditis elegans* suggests that during meiosis, colchicine affects the formation of the SC and disrupts the structure of the nuclear envelope [75]. Colchicine is a dinitro-sulfonamide herbicide and worm killer that binds selectively to α-tubulin and inhibits polymerization of microtubules. However, it is also a hydrophobic compound that may rupture membranes similar to detergents [75]. Oryzalin is a dinitro-aniline herbicide that has higher affinity to plant tubulins [13]. Trifluralin and N_2_O are believed to disrupt meiotic cytokinesis [73]. Trifluralin is a plant-specific dinitro-toluidine herbicide that also targets tubulin [76]. Trifluralin and colchicine may be applied directly to meiotic flower buds of *P*. Sogo Yukidian ‘V3’ (see Figure 1A,B) in lanolin paste (0.09–0.13% and 0.05–0.1%) to induce the formation of 2n gametes [77].

## 10. Conclusions

Meiotic polyploidization is an important tool for successful orchid breeding, and it may allow for the development of new and exciting orchid hybrids, as in *Phalaenopsis*. However, as an evolutionary process, it is complex and may involve genome fractionation, dysploidy, and homoeologous recombination, as seen in organisms such as *A. thaliana*, *A suecica*, and *B. napus*. Thus, its study in orchids may also allow for better understanding the mechanisms that shape genome evolution and that may be harnessed during horticultural selection. The integration of genomics and genome-editing tools may allow for the functional validation of gene function during orchid meiosis and the induction of valuable traits such as unreduced gamete formation.

## Figures and Tables

**Table 1:** Processes involved in meiotic polyploidization and karyotype evolution in orchids and plants in general.

Term	Description
*Aneuploidy*	Loss or gain of chromosomes relative to the normal chromosome complement [17]
*Allopolyploid*	Organism that combines two genomes or more deriving from related species [18]
*Autotetraploid*	A tetraploid plant formed directly from the merger of two unreduced gametes provided by the same diploid individual and formed via FDR [18]
*Allotetraploid*	A tetraploid formed from the merger of two unreduced gametes provided by different diploid plants and formed by FDR [18]
*Amphihaploid*	A haploid derived from unbalanced meiotic segregation in a hybrid [19]
*Autopolyploid*	Organism that combines two genomes or more deriving from the same species [18]
*Bivalent*	Pair of homologs physically linked at the end of metaphase I [18]
*Centric fission*	The break within the centromere of a single chromosome producing two whose raw ends can fuse after replication. Telomeric sequences may be added to the termini and two stable chromosomes are formed. Fission increases chromosome number and karyotype symmetry [20]
*Chiasmata*	The physical manifestation of reciprocal exchanges of DNA between non-sister chromatids (e.g., crossovers). Chiasmata maintain pairs of homologs linked at the end of metaphase I (as bivalents). At least one is required per bivalent to obtain well-balanced gametes and avoid aneuploidy [18]
*Chromosomal Inversion*	A structural change in chromosomes formed when two breaks are caused and the segment between them realigns and rejoins in the opposite orientation. To enable effective pairing in meiosis an inversion loop is formed. If a chiasma is formed within the loop the chromatids will form a dicentric bridge and an acentric fragment that is lost [18].
*Cytokinesis*	Also known as cytoplasm division. It is the formation of a cell wall in plant cells at the telophase stage. Cellular organelles such as mitochondria are partitioned between the new daughter cells [18].
*Descending dysploidy*	Evolutionary decrease in the base chromosome number (x). Also called polyploid drop and viewed as the mechanism that turns polyploids into functional diploids [21]
*Dyad*	A pair of cells instead of the usual four cells resulting from abnormal meiosis [4]
*First Division Restitution (FDR)*	A process in which a plant gamete is formed due to a defect in meiosis I. In these gametes there is neither recombination nor disjunction, thus parental heterozygosity is fully retained [19].
*Fractionation*	The loss of one or the other copy of a newly duplicated gene [22]
*Gene Dosage*	Relative expression of a gene product [18]. Polyploids avoid imbalances in gene dosage by retaining duplicates and allowing them to eventually develop new functions [23].
*Genome Dominance*	A process antagonistic to heterosis in which a subgenome gains relevance through dosage and functionalization and that may involve changes in chromatin marks and expression [23].
*Heterosis*	The unevolved retention of genes or cis regulatory elements as homoeologous pairs, it is antagonistic to dominance [23]. It is linked to vigor in wide hybrids and allopolyploids. It may manifest as the acceleration of somatic growth including the enlargement of organs [23].
*Homoeologous*	Non-homologous, often used to refer to meiotic pairing between non-homologous chromosomes. Homoeologous pairing reduces fertility [18].
*Homologous Recombination*	Exchange of DNA sequences with the same linear arrangement of genes between a paternal and maternal chromosome copy, also called a homologous pair [24].
*Karyotype*	Chromosome complement of an individual plant or species as described by number and morphology [4]
*Micronuclei*	Aberrant nuclei formed by the unequal distribution of chromosomes in daughter cells, often caused by unpaired chromosomes and common in tumor cell lines and meiotic cells of orchid hybrids [16,18].
*Multivalent*	Association during meiosis of more than two chromosomes whose homologous regions are synapsed by pairs [4]. Multivalent formation is detrimental to meiotic chromosome segregation and reduces fertility [18].
*Ploidy*	The basic chromosome set, often defined as ’x’. Thus, 2 x indicates that the organism has two basic sets of chromosomes. It is different from the number of chromosomes in zygotic cells defined as ’n´. For instance, bread wheat is a hexaploid in which 2n = 6x = 42, where x = 7 [18].
*Polyhaploid*	Haploid individual resulting from polyploid parents [4]
*Post-Polyploid Diploidization (PPD)*	A process of evolutionary modifications that transform a polyploid genome into a quasi-diploid one. It is mediated by homoeologous recombination leading to structural chromosomal changes including reduction of chromosome number [21].
*Robertsonian Fusion*	The fusion of two non-homologous chromosomes that gives rise to single chromosome with a single centromere [18,20]. As a result, the chromosome number is reduced, and the karyotype symmetry is increased [20].
*Second Division Restitution (SDR)*	A process in which a plant gamete is formed due to a defect in meiosis II. The exclusive separation of recombined homologs causes the formation of partially homozygous unreduced gametes [17].
*Somaclonal Variation*	Variation caused during tissue culture of orchids. Mutant plants can be distinguished by their morphological and physiological traits. It may be detected in the diploid karyotype of *Phalaenopsis*, but the specific chromosomal rearrangements are difficult to examine [25].
*Tetrad*	The four haploid products of meiosis [4]
*Tissue Culture*	General term for the aseptic growth of tissues, calls and organs *in vitro* [4]
*Univalents*	Homologs that fail to pair and form chiasmata between them at metaphase I. Their behavior at anaphase I is unpredictable and may not engage in successful division [18].
*Unreduced Gamete*	A gamete with the somatic chromosome complement [24].
*Whole Genome Duplication (WGD)*	Events of whole genome doublings (tetraploidizations) or whole genome triplications that lead to the formation of autotetraploid, allotetraploid and hexaploid plants [22].

**Table 2:** Genes from model plant *A. thaliana* reportedly involved in meiotic restitution, crossover formation and chromatid alignment of interest for breeding polyploid orchid hybrids.

Gene Name	Functional Features in the Literature and Databases ^1,2,3^	Original Locus ID According to TAIR and NCBI ^1,2^	Homologs of Interest in the Orchid Database *Orchidstra 2.0* ^4^	Horticultural Use
*ASY1NAPTIC1* (*ASY1*)	Encodes a protein with a *HORMA*-domain for recognition of chromatin states linked to DNA double-strand breaks, a peptidase domain, a *W*inged *H*elix (*WH*) DNA-binding domain and a *SWIRM*-domain for mediation of protein–protein interactions in the assembly of chromatin-protein complexes. ASY1 Participates in assembly of chiasmata and homologous chromosome pairing during meiosis. Mutants may show few chiasmata per nuclei.	*At1g67370*	*Phalaenopsis aphrodite* transcript *PATC148994* (E-Value: 1.32 × 10^−142^)	Heat-stress mediated disruption of synapsis and recombination. Formation of clonal gametes [44]
*CYCA1;2*/*TARDY ASYNCHRONOUS MEIOSIS* (*TAM*)	Encodes a N-terminal cyclin-like protein that may regulate cyclin dependent kinases (CDKs). Encodes a core cell cycle gene involved in meiosis II progression. Mutants develop dyads.	*At1g77390*	*Phalaenopsis modesta* transcript *PMTC011254* (E-Value: 7 × 10^−94^)	Formation of 2n gametes through SDR [45]
*DYAD/SWITCH1* (*SWI1*)	Encodes a protein that features a catalytic phosphatidylinositol-specific phospholipase C *X*-domain that is probably involved in signal transduction. Protein intervenes in chromatid cohesion establishment, in chromosome structure during male and female meiosis, and in axial element formation.	*At5g51330*	*Phalaenopsis lueddemanniana* transcript *PLTC046384* (E-Value: 4 × 10^−84^)	Development of unreduced gametes by FDR [43]
*ARABIDOPSIS THALIANA MAP KINASE 4* (*MAPK4*)	Encodes a protein with a conserved *M*itogen-*A*ctivated *P*rotein (*MAP*) kinase site. Required for male-specific meiotic cytokinesis. The mRNA is cell-to-cell mobile.	*At4g01370*	*Oncidium* Gower Ramsey transcript *OGTC022747* (E-Value: 6 × 10^−13^)	Formation of recombinant, SDR-type unreduced gametes [46]
*ARABIDOPSIS THALIANA MAP KINASE KINASE 6* (*MKK6*)	Encodes a kinase protein with an ATP-binding site. Phosphorylates MAPK4. Required for male meiotic cytokinesis.	*At5g56580*	*Phalaenopsis aphrodite* transcript *PATC137812* (E-Value: 5 × 10^−152^)	Formation of recombinant, SDR-type unreduced gametes [46]
*ARABIDOPSIS MUTS HOMOLOG 4* (*MSH4*)	Encodes a protein that features a core *MutS*, domain for DNA mismatch repair. It is involved in homologous chromosome segregation and meiotic mismatch repair during recombination. Mutants show low chiasmata formation.	*At4g17380*	*Phalaenopsis schilleriana* transcript *PSTC034674* (E-Value: 9 × 10^−36^)	Reduction of homoeologous recombination in polyploid hybrids, improved chromosome segregation [47]
*MMS and UV Sensitive 81* (*MUS81*)	Encodes a protein that features a WH-like DNA-binding domain for branch migration and transcriptional repression, and an ERCC4 domain for cleaving branched structures generated during DNA repair, replication, and recombination. Mutants show few bivalents.	*At4g30870*	*Phalaenopsis lueddemanniana* transcript *PLTC011379* (E-Value: 2.82 × 10^−69^)	Reduction of homoeologous recombination in polyploid hybrids, improved chromosome segregation [47]
*NPK1-ACTIVATING KINESIN 2*/*TETRASPORE* (*NACK2*/*TES*)	Encodes a protein with a kinesin motor domain and a P-loop NTPase domain. It is required for cytokinesis in pollen. In mutants, all four microspore nuclei remain within the same cytoplasm after meiosis.	*At3g43210*	*Cymbidium ensifolium* transcript *CETC000340* (E-Value: 6 × 10^−38)^	Formation of recombinant, SDR-type unreduced gametes [48]
*NPK1-RELATED PROTEIN KINASE 3* (*NP3*)	Encodes a protein with a Serine/Threonine kinase domain. Regulates microtubule organization. May regulate formation of the intersporal callose wall after male meiosis. Mutants may not complete meiotic cytokinesis.	*At3g06030*	*Phalaenopsis schilleriana* transcript *PSTC039874* (E-Value: 2 × 10^−41^)	Formation of recombinant, SDR-type unreduced gametes [46]
*OMISSION OF SECOND DIVISION 1/GIGAS CELL 1* (*OSD1/GIG1*)	Encodes a protein from the Polychome protein. It may work as a negative regulator of the activity of the anaphase-promoting complex/cyclosome (APC/C) ubiquitin ligase. Mutants produce diploid gametes by skipping the second meiotic division.	*At3g57860*	*Phalaenopsis schilleriana* transcript *PSTC034707* (E-Value: 7 × 10^−75^)	Formation of 2n gametes through SDR [45]
*PRECOCIOUS DISSOCIATION OF SISTERS 5B* (*PDS5B*).	The respective protein contains an armadillo (ARM)-like fold, consisting of a multi-helical fold comprised of two curved layers of alpha helices that allow for proteins and nucleic acids. The Arabidopsis genome contains five orthologues that are required for proper chromosome segregation at anaphase I.	*At1g77600*	*Phalaenopsis schilleriana* transcript *PSTC040504* (E-Value: 9 × 10^−30^)	Stabilization of meiosis in hybrid polyploids [22]
*RECOMBINATION 8/SYNAPTIC 1* (*REC8*/*SYN1*)	Encodes a RAD21-protein which may assemble as a hetero-tetramer that enables opening of SMC-kleisin rings. It is involved in chromosome condensation, pairing and segregation during meiosis. Responsible for cohesion between replicated sister chromatids.	*At5g05490*	*Phalaenopsis aphrodite* transcript *PATC192174* (E-Value: 9 × 10^−44^)	Heat-stress mediated disruption of synapsis and recombination. Formation of clonal gametes. Formation of unreduced gametes by FDR [44,49].
*REPLICATION PROTEIN A 1C* (*RPA1C*)	Encodes a factor known as the Replication Protein A-70kDa-DNA-binding subunit. Contains an *O*ligonucleotide/Oligosaccharide *B*inding motif, or *OB* fold, a five-stranded beta-sheet coiled to bind single-stranded DNA. This protein regulates DNA unwinding during replication, recombination, and repair. Mutants show incomplete synapsis and meiotic chromosome fragmentation.	*At5g45400*	*Phalaenopsis bellina* transcript *PBTC027818* (E-Value: 2.70 × 10^−128^)	Reduction of homoeologous recombination in polyploid hybrids, improved chromosome segregation [47]
*STRUCTURAL MAINTENANCE OF CHROMOSOMES 3/TITAN 7* (*SMC3/TTN7*)	May encode a member of the *S*tructural *M*aintenance of *C*hromosomes (*SMC*) family of proteins. These proteins share a five-domain structure, with globular N- and C-terminal domains separated by a coiled-coil segment with a globular ‘‘hinge’’ domain. The N-terminal domain contains a ‘Walker A’ nucleotide-binding domain, while the C-terminal domain contains a ‘Walker B’ motif and an ATP-binding cassette (ABC). SMC3 localizes to the axial elements of pachytene chromosomes. Heterozygous mutants show reduced cohesion along the arms.	*At2g27170*	*Phalaenopsis equestris* transcript *PETC035815* (E-Value: 3.57 × 10^−147^)	Stabilization of meiosis in hybrid polyploids [22]
*STRUCTURAL MAINTENANCE OF CHROMOSOMES 1/TITAN 8* (*SMC1/TTN8*)	May encode a SMC protein. Works together with SMC3 during the establishment of proper meiotic cohesion.	*At3g54670*	*Phalaenopsis bellina* transcript *PBTC023989* (E-Value: 3.25 × 10^−122^)	Stabilization of meiosis in hybrid polyploids [22]
*STRUCTURAL MAINTENANCE OF CHROMOSOMES 6B*/*HYPERSENSITIVE TO MMS, IRRADIATION AND MMC; MIM* (*SMC6B/MIM*)	May encode a protein part of the SMC5/6 complex. This complex promotes sister chromatid alignment and homologous recombination after DNA damage. Mutants produce unreduced gametes.	*At5g61460*	*Phalaenopsis equestris* transcript *PETC039012* (E-Value: 6.95 × 10^−111^)	Stabilization of meiosis in hybrid polyploids [22]
*SYNAPTONEMAL COMPLEX PROTEIN 1A* (*ZYP1A*)	May encode a myosin heavy chain-related protein. It may feature two coiled-coil domains and several sites for polar, basic, and acidic residues. It is involved in chromosome synapsis during meiosis I and localizes at the synaptonemal complex (SC), Mutants show improper or non-homologous synapsis.	*At1g22260*	*Phalaenopsis lueddemanniana* transcript *PLTC005471* (E-Value: 3.64 × 10^−24^)	Heat-stress mediated disruption of synapsis and recombination. Formation of unreduced gametes by FDR [44]

Abbreviations: FDR, first meiotic restitution; SDR, second meiotic restitution. ^1^ The Arabidopsis Information Resource (TAIR). Available online: https://www.arabidopsis.org (accessed on 6 March 2022). ^2^ The National Center for Biotechnology Information (NCBI). Available online: https://www.ncbi.nlm.nih.gov/nucleotide/ (accessed on 6 March 2022). ^3^ Universal Protein Resource (UniProt). Available online: https://www.uniprot.org/ (accessed on 6 March 2022). ^4^ Orchidstra 2.0, A Transcriptomics Resource for the Orchid Family. Available online: http://orchidstra2.abrc.sinica.edu.tw/orchidstra2/index.php (accessed on 13 March 2022).

## Data Availability

Not applicable.
